# A 25-year surveillance of disseminated Bacillus Calmette–Guérin disease treatment in children in Southern Iran

**DOI:** 10.1097/MD.0000000000009035

**Published:** 2017-12-29

**Authors:** Ali Amanati, Gholamreza Pouladfar, Mohammad Rahim Kadivar, Anahita Sanaei Dashti, Zahra Jafarpour, Sezaneh Haghpanah, Abdolvahab Alborzi

**Affiliations:** a Alborzi Clinical Microbiology Research Center, Shiraz University of Medical Sciences; b Hematology Research Center, Shiraz University of Medical Sciences, Shiraz, Iran.

**Keywords:** Bacillus Calmette–Guérin, children, disseminated disease, outcome, treatment, treatment efficacy

## Abstract

Disseminated Bacillus Calmette–Guérin (BCG) disease is one of the most serious complications of BCG vaccination, mainly among immunocompromised children with high morbidity and mortality.

Currently, there is no any consensus with regard to the standard regimen of antituberculosis (anti-TB) agents and duration of treatment in healthy or immunocompromised host in children. The aim of this study is to investigate the effect of various combination treatment strategies for disseminated BCG disease in children.

In this cross-sectional study, the outcome of 3 different combination protocols was investigated in 59 patients.

All patients were younger than 6 years old. Both possible immunocompetent and proven immunodeficient children were included in a period of 25 years (1991–2014) in a Nemazee referral teaching hospital.

The minimum age was 1 month and the maximum was 60 months. The average age of patients was 8 months (8.26 ± 9.73). Out of 59 cases, 32 (54.2%) were female and 27 (45.8%) were male. Based on the primary work up, 52.5% of cases were classified as definite immunodeficient and 47.5% were classified as possible immunocompetent. Overall mortality rate was 50.8%. Mortality rate of disseminated BCG disease in immunocompetent and immunodeficient children was 28.6% and 71%, respectively. The mortality rate was not statistically different between patients treated with different treatment protocols. These results were not affected by immune status and the type of immunodeficiency.

More than 2 anti-TB drugs combination will not change outcome of patient with disseminated BCG disease.

## 1 Introduction

### 1.1 Adverse events of BCG vaccine and its management

Bacillus Calmette–Guérin (BCG) vaccine is administered in developing countries to prevent severe form of tuberculosis and considered safe in healthy infants. Vaccine administration may be accompanied by local and systemic adverse events that may be mild to severe (Table 1).^[1]^ The most serious and rare complication of BCG vaccine is disseminated form.^[2–4]^ Dissemination occurs exclusively in immunocompromised host children after a birth dose of BCG vaccine^[5,6]^ with wide range of time frames.^[1,7–9]^ The reported incidence of disease is more than 1% in human immunodeficiency virus (HIV)-infected infants.^[1,10]^

**Table 1:**
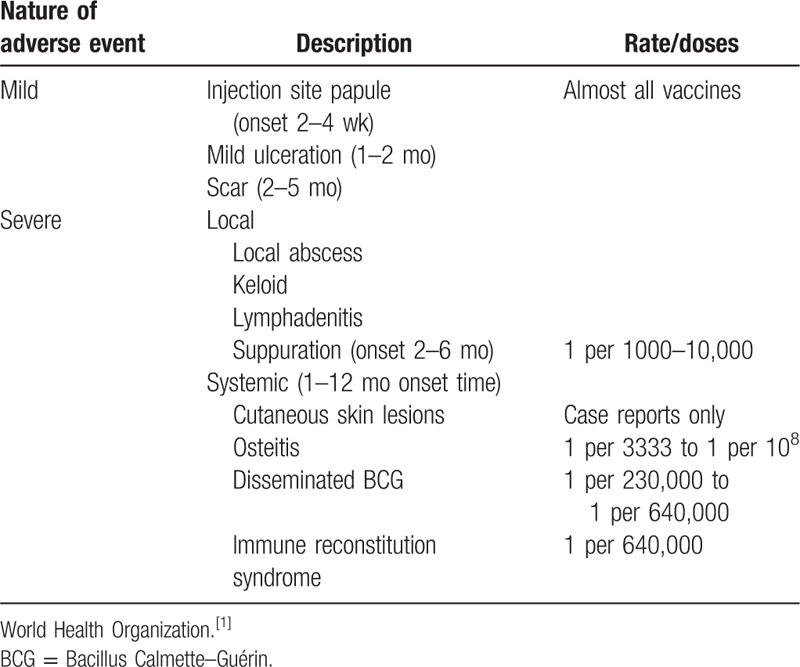
Summary of BCG vaccine adverse events and its estimated incidence.

The most common age of clinical presentation is early infancy (<12 months)^[7,11]^ but it can occur shortly after birth to 72 months of age.^[7,8,12]^ The mortality rate is high with the range of 40% to 80%.^[7,12,13]^

The best treatment strategy of disseminated BCG disease is still unknown. Various combination treatment protocols are currently in use. There is no strong evidence to support standard treatment approach due to rarity of disease, host factors, and unclear etiology of dissemination.

Decision making for choosing the most effective treatment protocol is usually based on experts’ opinion.^[14]^

This study tries to compare different treatment protocols to find the best treatment strategy for management of disseminated BCG disease in children.

## 2 Patients and methods

In this cross-sectional study, medical records of 59 patients with disseminated BCG disease were investigated. Participants included all patients with disseminated BCG disease who had been hospitalized in pediatric infectious disease ward since 1991 to 2014 in Nemazee hospital of Shiraz, Southern Iran. Cases were defined as definite disseminated BCG disease when *Mycobacterium bovis* was isolated by culture. There is evidence of dissemination and compatible clinical signs and symptoms exists based on working definition of disseminated BCG disease by Talbot et al.^[14]^

The questionnaire consisted of age, sex, immune status, diagnosis, treatment protocol, and outcome. Immunological work up included peripheral blood flowcytometery, serum immunoglobulin profile (IgM, IgG, IgE, and IgA), and dihydrorhodamine/nitroblue tetrazolium test. For suspected case, interferon-gamma receptor (IFNGR) deficiency was checked after availability of this test in Professor Alborzi Clinical Microbiology Research Center (PACMRC). To do this, the anticoagulated whole blood was stained with PE-labeled anti-CD119 antibody and incubated for 30 min in darkness at room temperature. After that RBCs were lyzed using lysis buffer for 10 min and the cell pellet was washed with phosphate buffered saline. The expression of IFNGR was measured on the lymphocyte population using flow cytometer.

Acid–fast bacilli (AFB, Ziehl–Neelsen) staining primarily was done on any suspected aspirate from bone marrow, liver, or lymph node. After establishment of polymerase chain reaction (PCR) technique in PACMRC for detection of *M bovis* BCG, PCR was done on any suspected sample in the presence of compatible clinical signs or symptoms of disseminated BCG disease (fever, weight loss, generalized lymphadenopathy, hepatomegaly, splenomegaly, or cytopenia), if the primary result of AFB staining was negative. Clinical sample aspirates (bone marrow, liver, or lymph node) was treated with sodium dodesyl sulfate and Tris–EDTA buffer solution with proteinase K and concurrently heated to 65°C for overnight. After boiling for 10 min, DNA was extracted with phenol–chloroform standard protocol. PCR assay was based on polymorphism in the direct repeat (DR) region of the Mycobacterium tuberculosis complexs that depends on the presence or absence of specific spacer region sequences between 2 DR sequences for differentiation between *M bovis* and *Mycobacterium tuberculosis*, as *M bovis* contains spacer regions 33 and 34, which are absent in *M tuberculosis*. In addition, *M bovis* BCG has 2 copies of spacer region 33 but only one of spacer region 34. We have used this PCR that could differentiate among *M bovis*, *M bovis* BCG, and *M tuberculosis*.^[15]^

Patients were treated with 2 (isoniazid and rifampin), 3 (isoniazid, rifampin, and ethambutol), 4 (isoniazid, rifampin, ethambutol, and clarithromycin), and 5 (isoniazid, rifampin, ethambutol, clarithromycin, and amikacin) antituberculosis (anti-TB) drugs randomly.

The patients who received 2, more than 2, and 4 anti-TB drugs were categorized as protocol 1, 2, and 3, respectively. According to the expert, duration of treatment in all patients was considered 24 months.

The study was approved by Medical ethics committee of Shiraz University of Medical Sciences.

### 2.1 Statistical analysis

Data were analyzed by SPSS version 21.0 (IBM Corp. IBM SPSS Statistics for Windows, Armonk, NY). Descriptive data were presented as mean, standard deviation, percentages, and appropriate charts. Comparison of qualitative data among different groups were done by chi-squared test and Fisher exact test. *P* value < .05 was considered statistically significant.

## 3 Results

Patients with disseminated BCG disease who had been admitted in pediatric infectious disease ward in Nemazee hospital were analyzed in this cross-sectional study (n = 59).

The minimum recorded age was 1 month and the maximum was 60 months (mean ± standard deviation: 8 months, 8.26 ± 9.73).

Out of 59 cases, 32 (54.2%) were female and 27 (45.8%) were male. Based on the primary work up, 52.5% of cases were definite immunodeficient and 47.5% were possible immunocompetent (given the incomplete diagnostic workup). The most common type of immunodeficiency disorders is severe combined immunodeficiency syndrome (SCID) followed by unspecified immunodeficiency (nonspecific changes of CD3, CD4, and CD8 count in peripheral blood flowcytometry; [International Statistical Classification of Diseases and Related Health Problems 10th Revision: D84.9]),^[16]^ chronic granulomatous disease, HIV, and histiocytosis, respectively. Demographic characteristics, immune status, diagnosis, treatment strategy, and outcome of 59 patients with disseminated BCG disease are shown in Table 2.

**Table 2:**
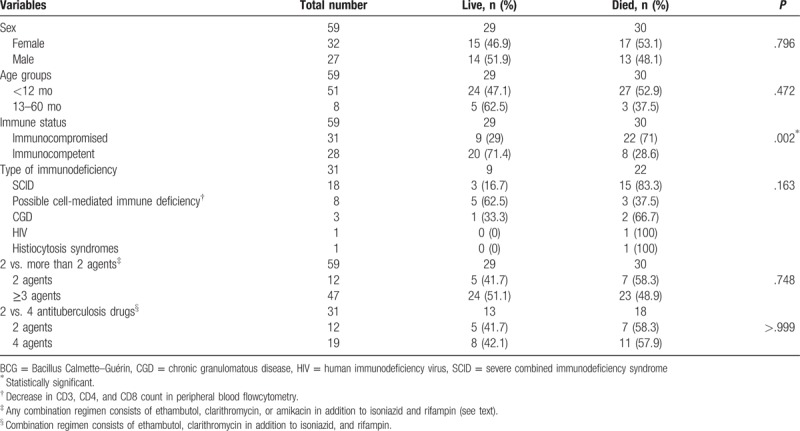
Demographic characteristics, immune status, diagnosis, treatment strategy, and outcome of 59 patients with disseminated BCG disease.

From the point of view of the clinical outcome, 71% of immunocompromised patients were died compared to 28.6% in immunocompetent group (odds ratio = 2.484, 95% confidence interval 1.326–4.652, *P* = .002). The outcome was not different between patients treated with 2 anti-TB drugs (isoniazid and rifampin, protocol 1) and patients treated with more than 2 anti-TB drugs (any combination regimen consists of ethambutol, clarithromycin, or amikacin in addition to isoniazid and rifampin, protocol 2; *P* = .748). Mortality rate also was not statistically different between patients treated with 2 anti-TB drugs (protocol 1) and patients treated with 4 anti-TB drugs (combination regimen consists of ethambutol, clarithromycin in addition to isoniazid, and rifampin, protocol 3; *P* > .999).

The same results obtained when different treatment protocols were compared in SCID patients as the most common type of immunodeficiency disorders with other types (for protocol 1 or 2 *P* > .522; and for protocol 1 or 3, *P* > .999). Data are summarized in Tables 3 and 4.

**Table 3:**
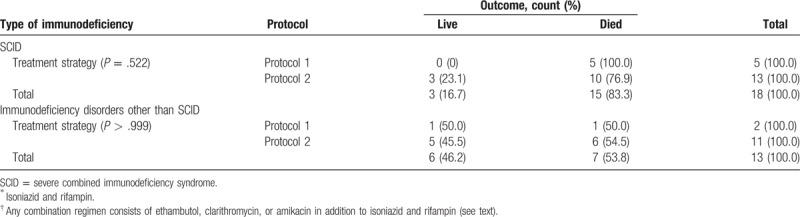
Treatment outcome in patients with SCID and other types of immunodeficiency disorders treated with 2 antituberculosis drugs protocol (1)^∗^ versus group of patients treated with more than 2 antituberculosis drugs protocols (2)^†^.

**Table 4:**
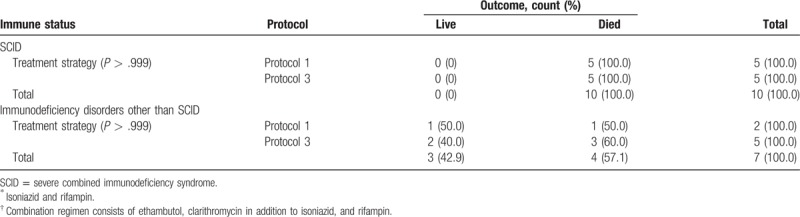
Treatment outcome in patients with SCID and other types of immunodeficiency disorders treated with 2 antituberculosis drugs protocol (1)^∗^ versus group of patients treated with 4 antituberculosis drugs protocol (3)^†^.

The possible effect of these protocols on outcome was also investigated in immunocompromise and immunocompetent patients separately. We found no significant difference in each group based on different protocols. For immunocompromise and immunocompetent patients who received protocol 1 or 2, *P* value was .639 and >.999, respectively. For patients who received protocol 1 or 3, *P* value was >.999 and >.999, respectively (Tables 5 and 6).

**Table 5:**
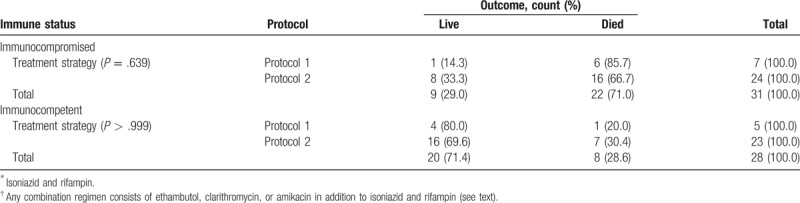
Treatment outcome in patients treated with 2 antituberculosis drugs protocol (1) ^∗^ versus group of patients treated with more than 2 antituberculosis drugs protocols (2)^†^.

**Table 6:**
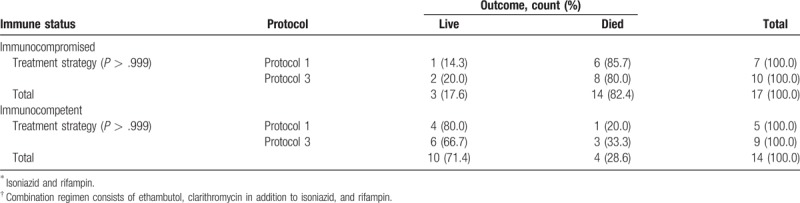
Treatment outcome in patients treated with 2 antituberculosis drugs protocol (1)^∗^ versus group of patients treated with 4 antituberculosis drugs protocol (3)^†^.

## 4 Discussion

Currently, various BCG strains are used in BCG vaccines for prevention of extrapulmonary tuberculosis (especially meningitis and miliary tuberculosis) in different parts of the worlds. Among them, Pasteur strain 1173 P2, Danish strain 1331, Glaxo strain 1077, and Tokyo strain 172 are the most common type in the world.^[17–21]^ Iranian newborn infants routinely receive BCG Pasture strain (BCG—Pasteur [1173 P2]) at birth. Recently, the susceptibility of *M bovis* in children with post-BCG lymphadenitis was investigated in PACMRC.^[22]^ Minimal inhibitory concentration of BCG Pasture strain and 24 clinical isolates were determined for first- and second-line anti-TB drugs. The results revealed that all BCG Pasture strain (original strain and clinical tested isolates) were sensitive to isoniazid and rifampin.^[2]^

There is a lack of unique treatment strategy for disseminated BCG disease among different centers regard to the best regimen as well as duration of treatment in literatures.^[5,7,8,12,14,23]^

Thus, given that all of BCG Pasture strains in use are sensitive to isoniazid and rifampin, we think that probably this combination may be effective and noninferior compare to other multidrug regimens (more than 2 agents). To evaluate this hypothesis, 3 different protocols in immunocompromise and immunocompetent children were investigated.

We did not find any significant effect of different treatment protocols containing 2 anti-TB agents versus those containing more than 2 agents on mortality (Tables 3–6).

Currently, SCID is the most common type of primary immunodeficiency syndrome (PID) among Iranian children (near 21.1%) based on the last published reports of Iranian Primary Immunodeficiency Registry.^[24]^ Interestingly, we found no significant effect of more than 2 anti-TB agents used for treatment of children with SCID on the outcome.

According to anti-TB susceptibility testing of our vaccine strain (BCG—Pasteur) which full susceptible to first-line anti-TB drugs including isoniazid and rifampin, adding more than 2 anti-TB drugs will have no impact on patient's outcomes.

## 5 Conclusions

Countries such as Iran where all newborn infants receive single dose of BCG vaccine at birth, many of those with cellular immunodeficiency syndromes (such as SCID) complicated with BCG vaccine. According to the results, isoniazid and rifampin for duration of 24 months are sufficient for treatment of disseminated BCG disease in children with PID.

## Acknowledgment

The authors would like to appreciate Ms. R. Farmani for English editing of the manuscript.

## References

[1] World Health Organization. The Vaccines, 2012. Available at: http://www.who.int/vaccine_safety/initiative/tools/BCG_Vaccine_rates_information_sheet.pdf. Accessed date: December 2016.

[2] Karimi A Tabatabaei SR Amanati A Multifocal dactylitis as a consequence of Bacillus Calmette–Guérin vaccination in a patient with severe combined immunodeficiency: a case report. Arch Pediatr Infect Dis 2015;3:e59841.

[3] Tabatabaei SR Amanati A Karimi A Diffuse hyperpigmented subcutaneous nodules as a primary manifestation of disseminated Bacillus Calmette–Guérin disease in young infants. Arch Pediatr Infect Dis 2015;3:e24744.

[4] Alborzi A Mostafavi N Retroperitoneal abscess due to disseminated Bacille Calmette–Guérin infection. Jpn J Infect Dis 2007;60:392–3. 18032842

[5] Shirvani F Karimi A Rajabnejad M BCG vaccination as a prevention strategy, threats and benefits. Arch Pediatr Infect Dis 2016;4:e30180.

[6] Alborzi A Sadeghi E Disseminated *Mycobacterium tuberculosis* in an infant with AIDS. Arch Iran Med 2011;14:296–8. 21726111

[7] Aelami MH Alborzi A Pouladfar G Post-vaccination disseminated Bacillus Calmette Guérin infection among children in Southern Iran. Jundishapur J Microbiol 2015;8:e25663. 26862381 10.5812/jjm.25663 PMC4740899

[8] Paiman SA Siadati A Mamishi S Disseminated *Mycobacterium bovis* infection after BCG vaccination. Iran J Allergy Asthma Immunol 2006;5:133–7. 17237565

[9] Bukhari E Alaklobi F Bakheet H Disseminated Bacille Calmette–Guérin disease in Saudi children: clinical profile, microbiology, immunology evaluation and outcome. Eur Rev Med Pharmacol Sci 2016;20:3696–702. 27649674

[10] Hesseling AC Johnson LF Jaspan H Disseminated Bacille Calmette–Guérin disease in HIV-infected South African infants. Bull World Health Organ 2009;87:505–11. 19649364 10.2471/BLT.08.055657 PMC2704039

[11] Mahmoudi S Khaheshi S Pourakbari B Adverse reactions to *Mycobacterium bovis* Bacille Calmette–Guérin vaccination against tuberculosis in Iranian children. Clin Exp Vaccine Res 2015;4:195–9. 26273579 10.7774/cevr.2015.4.2.195 PMC4524905

[12] Rezai MS Khotaei G Mamishi S Disseminated Bacillus Calmette–Guérin infection after BCG vaccination. J Trop Pediatr 2008;54:413–6. 18593737 10.1093/tropej/fmn053

[13] Azzopardi P Bennett CM Graham SM Bacille Calmette–Guérin vaccine-related disease in HIV-infected children: a systematic review [Review article]. Int J Tuberc Lung Dis 2009;13:1331–44. 19861003

[14] Talbot EA Perkins MD Silva SF Disseminated Bacille Calmette–Guérin disease after vaccination: case report and review. Clin Infect Dis 1997;24:1139–46. 9195072 10.1086/513642

[15] Yeboah-Manu D Yates MD Wilson SM Application of a simple multiplex PCR to aid in routine work of the mycobacterium reference laboratory. J Clin Microbiol 2001;39:4166–8. 11682550 10.1128/JCM.39.11.4166-4168.2001 PMC88507

[16] ICD List. ICD-10 Diagnosis Code D84.9, Immunodeficiency, unspecified, 2017. Available at: http://icdlist.com/icd-10/D84.9. Accessed date: March 2017.

[17] Ritz N Tebruegge M Connell TG Susceptibility of *Mycobacterium bovis* BCG vaccine strains to antituberculous antibiotics. Antimicrob Agents Chemother 2009;53:316–8. 18955515 10.1128/AAC.01302-08 PMC2612166

[18] Dubos R Pierce C Schaefer W Differential characteristics in vitro and in vivo of several substrains of BCG. III. Multiplication and survival in vivo. Am Rev Tuberc 1956;74:683–98. 13372954 10.1164/artpd.1956.74.5.683

[19] Luca S Mihaescu T History of BCG vaccine. Maedica (Buchar) 2013;8:53–8. 24023600 PMC3749764

[20] Behr MA Small PM A historical and molecular phylogeny of BCG strains. Vaccine 1999;17:915–22. 10067698 10.1016/s0264-410x(98)00277-1

[21] World Health Organization. BCG vaccine: WHO position paper. Wkly Epidemiol Rec 2004;4:25–40. 14768305

[22] Jasem Mohamadi JP Alborzi A Pakzad I Anti-tuberculosis drugs sensitivity of BCG pasture strain isolated from lymphadenitis of children after vaccination by BCG vaccine. Int J Adv Biotechnol Res 2017;8:828–34.

[23] Shahmohammadi S Saffar MJ Rezai MS BCG-osis after BCG vaccination in immunocompromised children: case series and review. J Pediatr Rev 2014;2:62–74.

[24] Aghamohammadi A Mohammadinejad P Abolhassani H Primary immunodeficiency disorders in Iran: update and new insights from the third report of the national registry. J Clin Immunol 2014;34:478–90. 24659230 10.1007/s10875-014-0001-z

